# Administration of α-Klotho Does Not Rescue Renal Anemia in Mice

**DOI:** 10.3389/fped.2022.924915

**Published:** 2022-06-23

**Authors:** Min Young Park, Carole Le Henaff, Despina Sitara

**Affiliations:** ^1^Department of Molecular Pathobiology, NYU College of Dentistry, New York, NY, United States; ^2^Medicine, NYU School of Medicine, New York, NY, United States

**Keywords:** chronic kidney disease, Klotho, anemia, iron deficiency (anemia), inflammation

## Abstract

Renal anemia is a common complication in chronic kidney disease (CKD), associated with decreased production of erythropoietin (EPO) due to loss of kidney function, and subsequent decreased red blood cell (RBC) production. However, many other factors play a critical role in the development of renal anemia, such as iron deficiency, inflammation, and elevated fibroblast growth factor 23 (FGF23) levels. We previously reported that inhibition of FGF23 signaling rescues anemia in mice with CKD. In the present study we sought to investigate whether α-Klotho deficiency present in CKD also contributes to the development of renal anemia. To address this, we administered α-Klotho to mice with CKD induced by an adenine-rich diet. Mice were sacrificed 24 h after α-Klotho injection, and blood and organs were collected immediately post-mortem. Our data show that α-Klotho administration had no beneficial effect in mice with CKD-associated anemia as it did not increase RBC numbers and hemoglobin levels, and it did not stimulate EPO secretion. Moreover, α-Klotho did not improve iron deficiency and inflammation in CKD as it had no effect on iron levels or inflammatory markers. Interestingly, Klotho supplementation significantly reduced the number of erythroid progenitors in the bone marrow and downregulated renal *Epo* and *Hif2*α mRNA in mice fed control diet resulting in reduced circulating EPO levels in these mice. In addition, Klotho significantly decreased intestinal absorption of iron in control mice leading to reduced serum iron and transferrin saturation levels. Our findings demonstrate that α-Klotho does not have a direct role in renal anemia and that FGF23 suppresses erythropoiesis in CKD *via* a Klotho-independent mechanism. However, in physiological conditions α-Klotho appears to have an inhibitory effect on erythropoiesis and iron regulation.

## Introduction

Anemia is a common complication of chronic kidney disease (CKD) associated with poor outcomes such as reduced quality of life and increased cardiovascular disease, hospitalizations, cognitive impairment, and mortality (1). The kidney is the main source of the circulating protein erythropoietin (EPO), which is responsible for stimulating erythropoiesis. As kidney disease progresses, the kidneys are unable to produce sufficient amount of EPO and the prevalence of anemia increases, affecting nearly all patients with stage 5 CKD / end stage renal disease (ESRD) (1). However, the cause of anemia in CKD is multifactorial and includes not only insufficient EPO production but also pro-inflammatory cytokine activation and iron deficiency, among others.

Renal anemia is closely linked to iron-deficiency, which is due to both severely reduced or absent iron stores (absolute iron deficiency) and insufficient iron availability despite adequate iron stores (functional iron deficiency) (2–6). Functional iron deficiency is due to underlying inflammation which stimulates hepatic secretion of hepcidin that inhibits iron absorption in the gut and iron release from body stores (3, 5, 7). Renal anemia is typically normocytic, normochromic, and hypoproliferative due to reduced EPO activity in the bone marrow. However, the presence of iron deficiency in CKD results in microcytic and hypochromic erythrocytes (8–10).

Renal anemia is typically managed by the use of agents that stimulate erythropoiesis (ESAs) to activate Epo receptors (Epo-Rs) on erythroid progenitor cells directly or indirectly. Despite their benefits in anemia management, ESAs are linked to several adverse effects such as worsened hypertension, thromboembolic complications, tumor growth, as well as resistance to Epo therapy itself (1, 3, 11–13). In addition, ESAs do not improve adverse outcomes associated with anemia, such as mortality, cardiovascular events, left ventricular hypertrophy, or progression of kidney disease (1). Thus, there is a need to discover mechanisms that would stimulate erythropoiesis with fewer side effects.

Fibroblast growth factor 23 (FGF23) is a phosphaturic hormone produced by bone that regulates phosphate homeostasis by inducing phosphate excretion through its binding to the Klotho-FGFR complex. CKD is characterized by disordered mineral ion homeostasis, associated with marked elevation of FGF23 and Klotho deficiency (14, 15). In our previous studies we showed that FGF23 is a negative regulator of erythropoiesis and deletion of *Fgf23* in mice stimulates erythropoiesis (16). Similarly, disruption of FGF23 signaling by inactivation of *Klotho* results in increased red blood cell production (17). Importantly, we reported that inhibition of FGF23 signaling by the use of an antagonist rescues anemia, and ameliorates iron deficiency and inflammation in mice with CKD (18).

Growing evidence suggests that α-Klotho is a significant biomarker for CKD (14, 19) and a pathogenic factor in CKD progression (20, 21). Moreover, overexpression of *Klotho* or addition of exogenous recombinant Klotho increased renal EpoR protein and mRNA (22, 23). A role for Klotho in iron metabolism has also been reported by showing that serum iron overload decreases renal *Klotho* expression, whereas iron chelation suppresses angiotensin II-induced downregulation of *Klotho* (24). In the present study, we sought to investigate whether Klotho deficiency contributes to the development of renal anemia and iron deficiency and whether administration of Klotho can improve these conditions.

## Materials and Methods

### Animal Study

Six-week-old C57/B6J male mice were purchased from The Jackson Laboratory (Bar Harbor, ME, USA) and housed at New York University (NYU) College of Dentistry Animal Facility, where they were kept on a light/dark (12 h/12 h) cycle at 23°C, and received food (standard chow) and water *ad libitum*. Upon arrival, mice were acclimatized for 1 week, after which they were randomly assigned to two groups fed either control diet (TD 200790, Harlan Teklad, Madison, WI) or 0.2% adenine diet (TD 200791) for 8 weeks, as described in (25). After 8 weeks, control and adenine-fed mice were randomly assigned to 4 groups to receive intraperitoneally either saline (vehicle) or 10 μg/kg of recombinant mouse α-Klotho protein (R&D systems, Minneapolis, MN, USA). Groups: Control diet + Klotho, Control diet + Vehicle, Adenine diet + Klotho, and Adenine diet + Vehicle. Mice were sacrificed 24 hrs after Klotho or saline injection. The institutional animal care and use committee (IACUC) at New York University approved the animal studies.

### Blood, Serum and Tissue Collection

Mice were immediately necropsied after euthanasia and blood was collected by cardiac puncture. Whole blood was collected in Microtainer^®^ Blood Collection Tubes with K_2_EDTA (Becton, Dickinson and Company, Franklin Lakes, NJ) and complete blood count was performed using the VetScan HM5 Hematology Analyzer (ABAXIS, Union City, CA). For serum, blood was obtained in separate Microtainer^®^ serum separator tube (Becton, Dickinson and Company, Franklin Lakes, NJ) and centrifuged at 1,800 x g for 15 min. Liver, spleen, kidneys, intestine, bones (diaphysis), and bone marrow cells obtained from femora and tibiae were collected, snap-frozen in liquid nitrogen, and stored at −80°C until further use.

### Serum and Tissue Measurements

Serum and urinary phosphorus and creatinine levels were determined by colorimetric measurements using the Stanbio Phosphorus Liqui-UV^®^ and Stanbio Direct Creatinine LiquiColor^®^ Test reagents, respectively (Stanbio Laboratory, Boerne, TX). Serum FGF23 levels were measured using mouse FGF23 Intact and C-terminal ELISA assays (Quidel Corporation/Immutopics International, San Clemente, CA, USA). Serum iron and transferrin saturation were measured using the Iron-TIBC kit from Pointe Scientific (Canton, MI, USA). Tissue iron content was determined by colorimetric iron assay kit (Abcam, Waltham, MA, USA). Serum erythropoietin (EPO) and parathyroid hormone (PTH) levels were measured using the Rat/Mouse EPO Quantikine (R&D Systems, Minneapolis, MN, USA), and MicroVue Mouse PTH 1-84 (Quidel Corporation/Immutopics International, San Clemente, CA, USA) ELISA assays, respectively.

### Colony-Forming Unit Assay

A colony-forming unit assay was performed for bone marrow (BM) cells collected from both tibiae and femoral bones. The cells were plated in methyl cellulose medium (MethoCult M3231, Stemcell Technologies Inc., Vancouver, BC, Canada) at 2 × 10^5^ cells/mL and incubated at 37°C and 5% CO_2_. After 12 days of incubation, the colonies were scored based on morphology as burst-forming unit-erythoid (BFU-E) under a Nikon TMS inverted phase contrast microscope (Nikon Instruments, Melville, NY).

### RNA Isolation, Reverse Transcription, and Real-Time Quantitative PCR Analysis

Total RNA was extracted from kidneys, liver, duodenum, and bone marrow using Trizol (Ambion; Life Technologies, Carlsbad, CA, USA) according to the manufacturer's protocol (Molecular Research Center, Cincinnati, OH, USA). cDNA was synthesized using the High Capacity cDNA Reverse Transcription Kit, as described by the manufacturer (Applied Biosystems; Thermo Fisher Scientific, Waltham, MA, USA), and amplified by quantitative PCR (qPCR) using the PerfeCTa SYBR Green SuperMix (Quanta Biosciences, Gaithersburg,MD, USA). All primers used in this study are listed in Supplementary Table 1. mRNA levels were normalized to the housekeeping gene (*Hprt*) in the same RT sample. The relative transcript expression of a gene is given as ΔCt = Ct_target_-Ct_reference_. The fold change in gene expression, as compared to control mice, was determined as 2^ΔΔCt^ values (ΔΔCt = ΔCt_treated_-ΔCt_control_).

### Statistics

All data were analyzed by two-way analysis of variance (ANOVA) to determine the effect of diet (control vs. adenine diet) and/or treatment (saline vs. recombinant Klotho protein) using GraphPad Prism version 8.0 for Windows (GraphPad Software, San Diego, CA, USA). Outliers were identified and removed using the ROUT method prior to statistical analysis. Bonferroni correction was used to adjust for multiple pairwise comparison. All data were expressed at mean ± SD. *P* < 0.05 were considered significant.

## Results

### Evaluation of Renal Insufficiency in Mice Fed Adenine Diet

**Figure 1:**
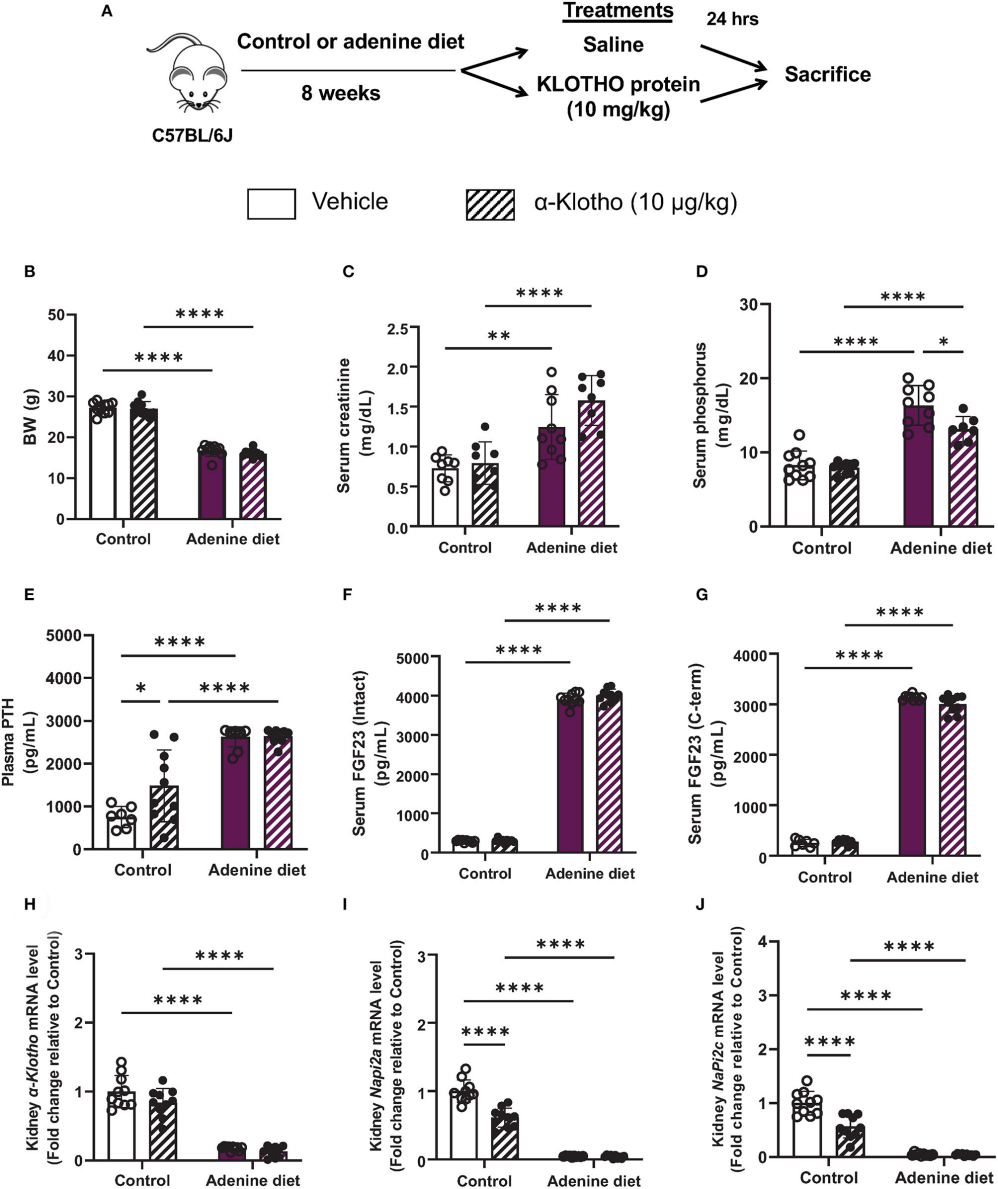
Validation of chronic kidney disease by adenine diet in mice. **(A)** A schematic of the experimental design. C57BL/6J male mice were fed control or 0.2% adenine diet for 8 weeks. After 8 weeks, mice in each group were further divided into 2 groups and were injected with either saline or mouse recombinant Klotho protein (10 μg/kg) 24 h before sacrifice. **(B)** Body weight at the end of experiment. **(C,D)** Serum concentration of **(C)** creatinine and **(D)** phosphorus. **(E)** Plasma PTH levels measured by ELISA. **(F)** Intact and **(G)** C-terminal FGF23 levels in serum measured by ELISA. **(H–J)** Quantitative real-time RT-PCR for renal **(H)** α-*Klotho*, **(I)** *Napi2a*, and **(J)** *Napi2c* expression. Data are expressed as fold change (2^−ΔΔCt^) relative to housekeeping gene *Hprt*. Samples were measured in duplicates. Data are represented as mean ± SD. (*n* = 7–10 per group). *P < 0.05, **P < 0.01, ****P < 0.0001 compared to Control.

In the present study, we used a non-surgical mouse model of renal failure induced by a diet rich in adenine. After developing renal failure, adenine-fed and control mice were randomized to receive either Klotho protein or vehicle. A schematic of the experimental design is shown in Figure 1A. Consistent with other studies (26–29), mice fed adenine had significantly reduced body weight and kidney weight in comparison to mice fed control diet (Table 1). However, the total kidney size, as assessed by the kidney weight to body weight ratio, was not different between adenine-fed and control mice (Table 1). Kidney function was appropriately impaired following consumption of adenine, as determined by elevated serum creatinine concentration (Table 2) and decreased phosphate urinary excretion (Supplementary Figure 1). Similar to CKD patients (30, 31), the adenine group also developed significant hyperphosphatemia, secondary hyperparathyroidism, and severely elevated FGF23 (Table 2). Moreover, we found that renal expression of *Klotho* and the sodium phosphate transporters *NaPi2a* and *NaPi2c* was significantly decreased in mice fed adenine diet compared to control mice (Figures 1H–J), in agreement with previous reports (32–37). Survival was identical between the two groups of mice (adenine-fed and control-fed) during our study. Together, these findings clearly demonstrate the development of kidney disease in our mice following consumption of adenine diet.

**Table 1:** Body and organ weights.

	**Control diet**	**Adenine diet**
Body weight (g)	27.234 ± 1.460	16.791 ± 1.411****
Kidney weight (g)	0.314 ± 0.022	0.192 ± 0.044****
Kidney weight/body weight	1.150 ± 0.054	1.063 ± 0.066
Spleen weight (g)	0.070 ± 0.007	0.050 ± 0.008****
Spleen weight/body weight	0.255 ± 0.020	0.296 ± 0.039
Liver weight (g)	1.296 ± 0.108	0.696 ± 0.069****
Liver weight/body weight	4.713 ± 0.386	4.137 ± 0.249***

*Values are means ± SD*.

*** *p < 0.001 (p = 0.0008)*.

**** *p < 0.0001 compared with control diet*.

**Table 2:** Biochemical parameters.

	**Control diet**	**Adenine diet**
Serum phosphorus (mg/dl)	8.256 ± 1.936	16.336 ± 2.673****
Serum creatinine (mg/dl)	0.725 ± 0.169	1.245 ± 0.404**
Serum PTH (pg/ml)	756.958 ± 245.057	2630.740 ± 232.841****
Serum intact FGF23 (pg/ml)	293.287 ± 27.669	3911.919 ± 154.716****
Serum C-term FGF23 (pg/ml)	252.610 ± 70.493	3134.364 ± 53.734****

*Values are means ± SD*.

** *p < 0.01 (p = 0.0089)*.

**** *p < 0.0001 compared with control diet*.

Our data also show that 24 h after Klotho administration, there was no effect of Klotho on body weight (Figure 1B) and organ weight or size (Supplementary Figure 2). Biochemical parameters such as serum creatinine, PTH, and FGF23 were also not affected by Klotho treatment and remained elevated in adenine-fed mice (Figures 1C,E–G). However, the prevailing hyperphosphatemia in these mice was significantly reduced after Klotho treatment (Figure 1D). In control mice, Klotho treatment increased serum PTH (Figure 1E) and it was capable of reducing *Napi2a* and *Napi2c* expression (Figures 1I,J), consistent with previous reports showing a direct interaction between circulating Klotho and Npt2a to suppress renal phosphate reabsorption (38, 39).

### Klotho Administration Does Not Rescue Renal Anemia

**Figure 2:**
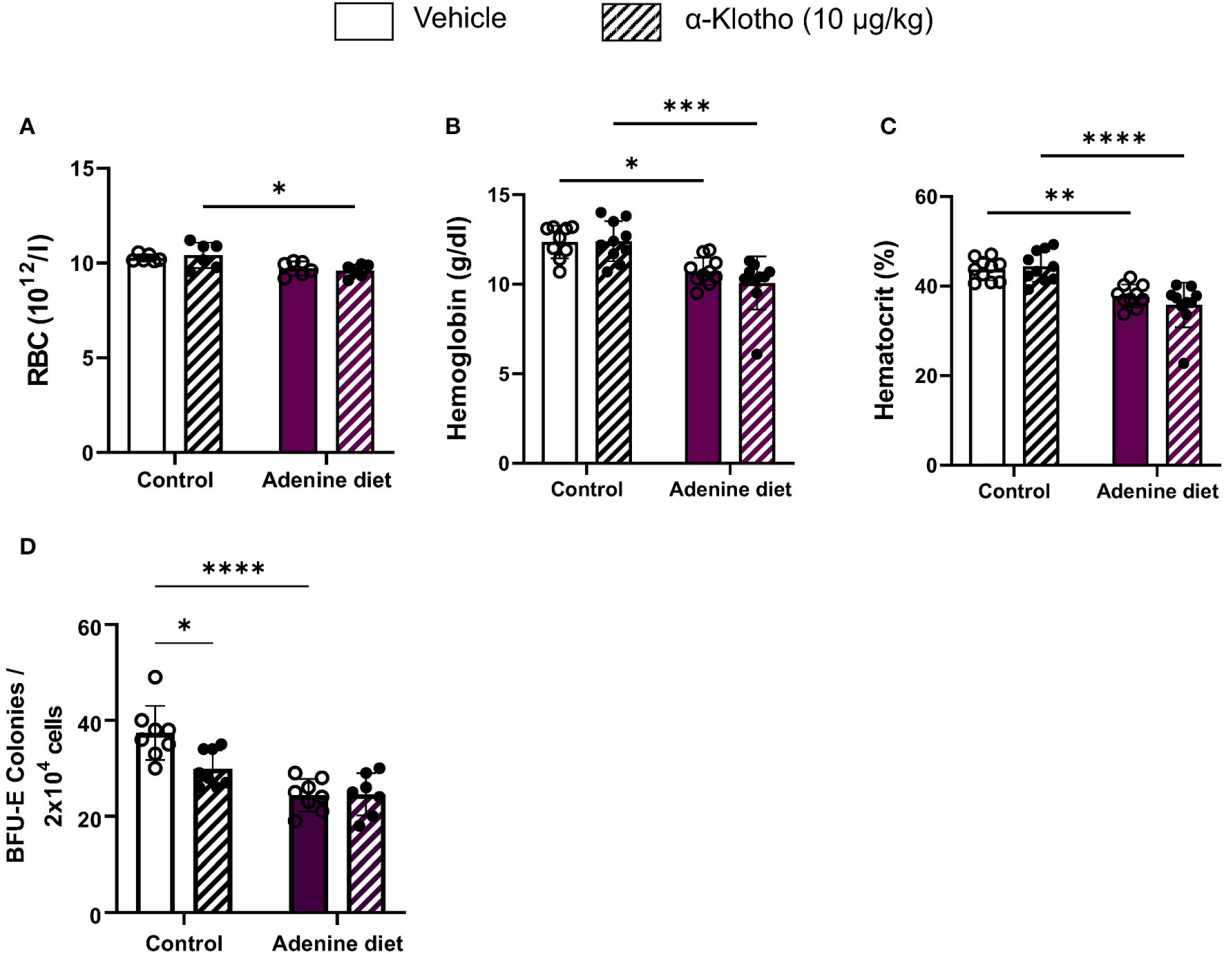
Effect of adenine diet and Klotho administration on circulating red blood cell parameters and bone marrow erythroid progenitor cells. **(A–C)** Whole blood was collected and analyzed by complete blood count for circulating red blood cell parameters. **(A)** Red Blood Cells (RBCs), **(B)** Hemoglobin (Hgb), **(C)** Hematocrit (Hct). **(D)** Femoral and tibial bone marrow cells were isolated and cultured for 12 days on methyl cellulose medium and counted for erythroid progenitor cells, burst forming unit erythroid (BFU-E). Data are represented as mean ± SD (*n* = 7–10 per group). *P < 0.05, **P < 0.01, ***P < 0.001, ****P < 0.0001 compared to Control.

Anemia is a common complication of CKD that develops primarily due to insufficient secretion of EPO by the diseased kidneys. Moreover, low levels of hemoglobin and hematocrit have been reported in both CKD patients and mice with 5/6 nephrectomy (40–46). Similarly, we found that mice fed adenine diet exhibited anemia, as it was determined by low hemoglobin, hematocrit, and serum EPO levels (Table 3), compared to mice fed control diet. Furthermore, we assessed the presence of erythroid progenitors in the bone marrow of control and adenine-fed mice by colony-forming unit (CFU) assay. Erythroid progenitors, termed burst-forming unit-erythroid (BFU-E), are detected by the formation of discrete erythroid colonies *in vitro*. As shown in Figure 2D, bone marrow (BM) cells from adenine-fed mice generated significantly less BFU-E colonies *in vitro* compared to BM cells from the control group, suggesting that BM cells from adenine-fed mice contain less functional erythroid progenitors resulting in anemia.

**Table 3:** Hematological parameters.

	**Control diet**	**Adenine diet**
RBC (10^12^/L)	9.884 ± 0.549	10.054 ± 0.555
Hemoglobin (g/dL)	12.563 ± 0.707	10.438 ± 0.529**^1^
Hematocrit (%)	43.730 ± 2.402	37.852 ± 2.505**^2^
MCV (fl)	44.000 ± 0.919	37.700 ± 0.823****
MCH (pg)	12.520 ± 0.266	10.650 ± 0.284****
RDW (%)	18.520 ± 0.391	19.800 ± 0.485****
WBC (10^9^/L)	1.749 ± 0.364	3.163 ± 1.323*^1^
Neutrophils (10^9^/L)	0.505 ± 0.342	1.833 ± 0.983**^3^
Lymphocytes (10^9^/L)	1.269 ± 0.221	0.692 ± 0.231*^2^
Platelets (10^9^/L)	386.667 ± 46.530	608.889 ± 88.373****
EPO (pg/ml)	333.722 ± 93.820	115.571 ± 28.936****
Serum iron (μg/dl)	74.059 ± 9.738	43.806 ± 12.091***

*Values are means ± SD*.

* ^1^p < 0.05 (p = 0.0485) / *^2^p < 0.05 (p = 0.0441).

** ^1^p < 0.01 (p = 0.0022) / **^2^p < 0.01 (p = 0.0036) / **^3^p < 0.01 (p = 0.0055).

*** *p < 0.001 (p = 0.0003)*.

**** *p < 0.0001 compared with control diet*.

Work from our lab has shown that inhibition of FGF23 signaling rescues renal anemia in the 5/6 nephrectomy mouse model (18). Here, we investigated whether amelioration of Klotho deficiency by Klotho protein administration can improve anemia in mice with adenine-induced CKD. Our data show that a single injection of Klotho protein did not have any effect on red blood cells (RBCs) (Figure 2A), hemoglobin (Figure 2B), or hematocrit levels (Figure 2C) in either adenine-fed or control mice. Furthermore, treatment with Klotho did not affect the number of BFU-E colonies formed *in vitro* in adenine-fed mice (Figure 2D). However, in control mice Klotho reduced the number of erythroid progenitors (Figure 2D).

**Figure 3:**
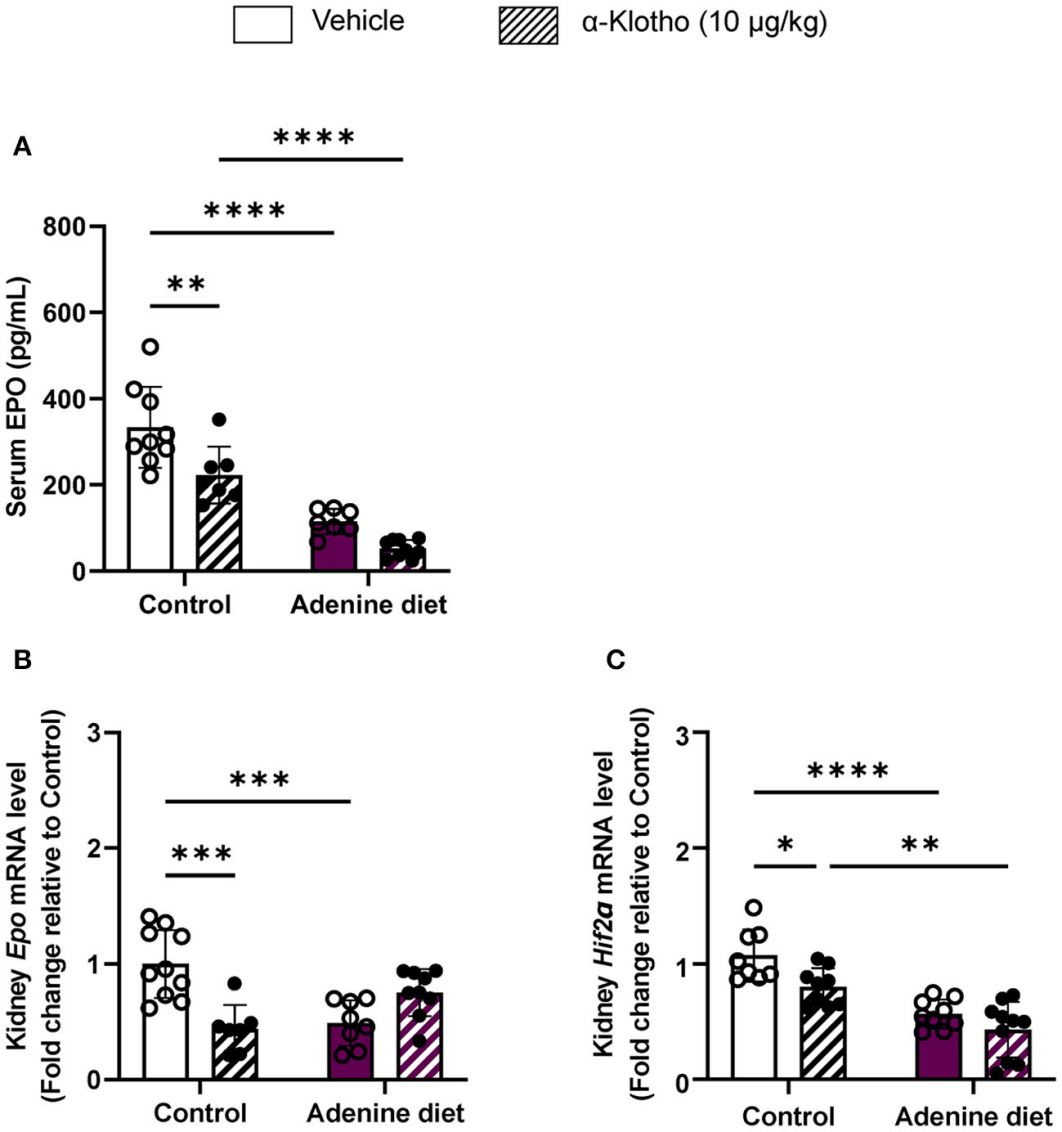
Regulation of erythropoietin production by Klotho administration. C57BL/6J male mice were fed control diet or 0.2% adenine diet for 8 weeks, and further divided into 2 groups for administration of either saline or mouse recombinant Klotho protein (10 μg/kg) 24 h prior to sacrifice. **(A)** Serum erythropoietin (EPO) levels measured by ELISA. **(B,C)** Quantitative real-time RT-PCR for expression of **(B)** renal *Epo*, and **(C)** renal *Hif-2*α. Data are expressed as fold change (2^−ΔΔCt^) relative to housekeeping gene *Hprt*. Data are represented as mean ± SD (*n* = 7–10 per group). *P < 0.05, **P < 0.01, ***P < 0.001, ****P < 0.0001 compared to Control.

Consistent with the decrease in circulating EPO levels, renal *Epo* mRNA expression was significantly suppressed in adenine-fed mice (Figure 3B) due to significant down-regulation of the hypoxia-inducible transcription factor (HIF) *Hif2*α (Figure 3C). We further evaluated the effect of Klotho administration on erythropoietin production in mice with adenine-induced CKD compared to mice fed control diet. Klotho treatment did not affect Epo secretion in adenine-fed mice, as it was evident by the unchanged levels of serum EPO (Figure 3A), as well as *Epo* and *Hif2*α expression in the kidneys (Figures 3B,C). Conversely, circulating EPO levels and renal Epo mRNA expression were significantly reduced by Klotho in control mice (Figures 3A,B). This reduction in *Epo* was due to decreased *Hif2*α mRNA expression in the kidneys (Figure 3C). Taken together, our data suggest that Klotho administration does not rescue renal anemia but in physiological conditions Klotho appears to suppress early stages of erythropoiesis.

### Klotho Administration Does Not Improve Iron Deficiency and Inflammation in CKD Mice

**Figure 4:**
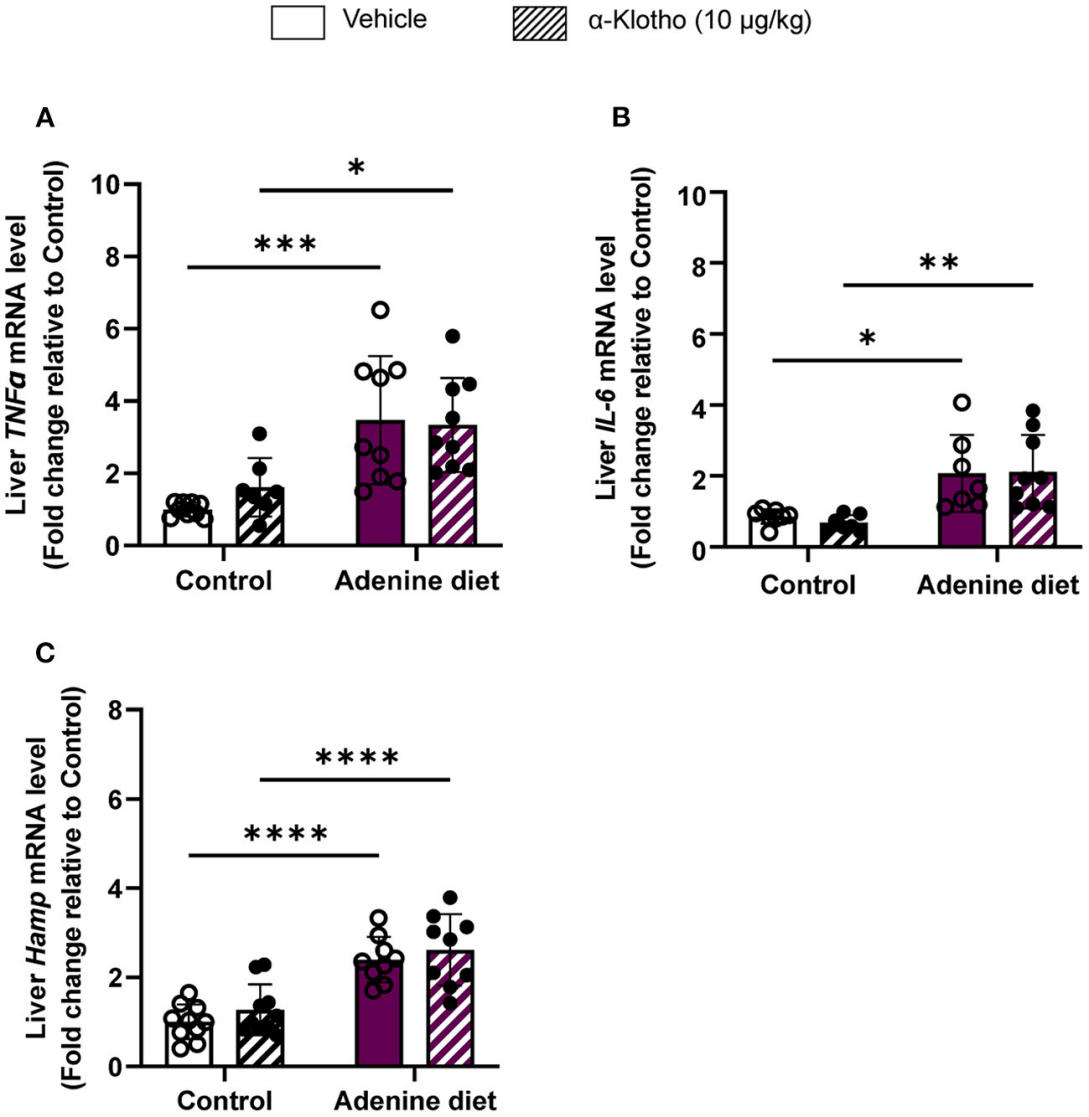
Effect of Klotho administration on inflammation in CKD. C57BL/6J male mice were fed control diet or 0.2% adenine diet. After 8 weeks, mice in each group were further divided into 2 groups and were injected with either saline or mouse recombinant Klotho protein (10 μg/kg) 24 h prior to sacrifice. Liver tissue samples were collected at the end of the experiment and relative mRNA expression was determined by quantitative real-time RT-PCR for **(A)** *TNF*α, **(B)** *IL-6*, and **(C)** *hepcidin (Hamp)*. Data are represented as mean ± SD (*n* = 7–10 per group). *P < 0.05, **P < 0.01, ***P < 0.001, ****P < 0.0001 compared to Control.

Iron is an essential element for hemoglobin synthesis and normal differentiation and proliferation of erythroid progenitor cells. Iron homeostasis is achieved by tight regulation between duodenal iron absorption and macrophage iron recycling. Hepcidin, a liver-secreted protein, is a master regulator of iron metabolism controlling the release of iron from the stores into the circulation (47, 48). Hepcidin is upregulated during inflammation, particularly in chronic inflammatory conditions such as CKD, leading to anemia, also known as anemia of chronic disease or anemia of inflammation (49, 50). Patients with CKD have elevated levels of pro-inflammatory cytokines (e.g., IL-6, IL-1β, TNFα, etc.) leading to upregulation of hepcidin (51). Consistent with the patient data, inflammatory markers, TNFα and IL-6, were significantly increased in the liver of adenine-fed mice (Figures 4A,B) compared to control mice, resulting in upregulation of hepcidin (*Hamp*) mRNA expression (Figure 4C). Klotho administration did not have any effect on these inflammatory markers or hepcidin expression (Figures 4A–C) in either adenine or control groups.

**Figure 5:**
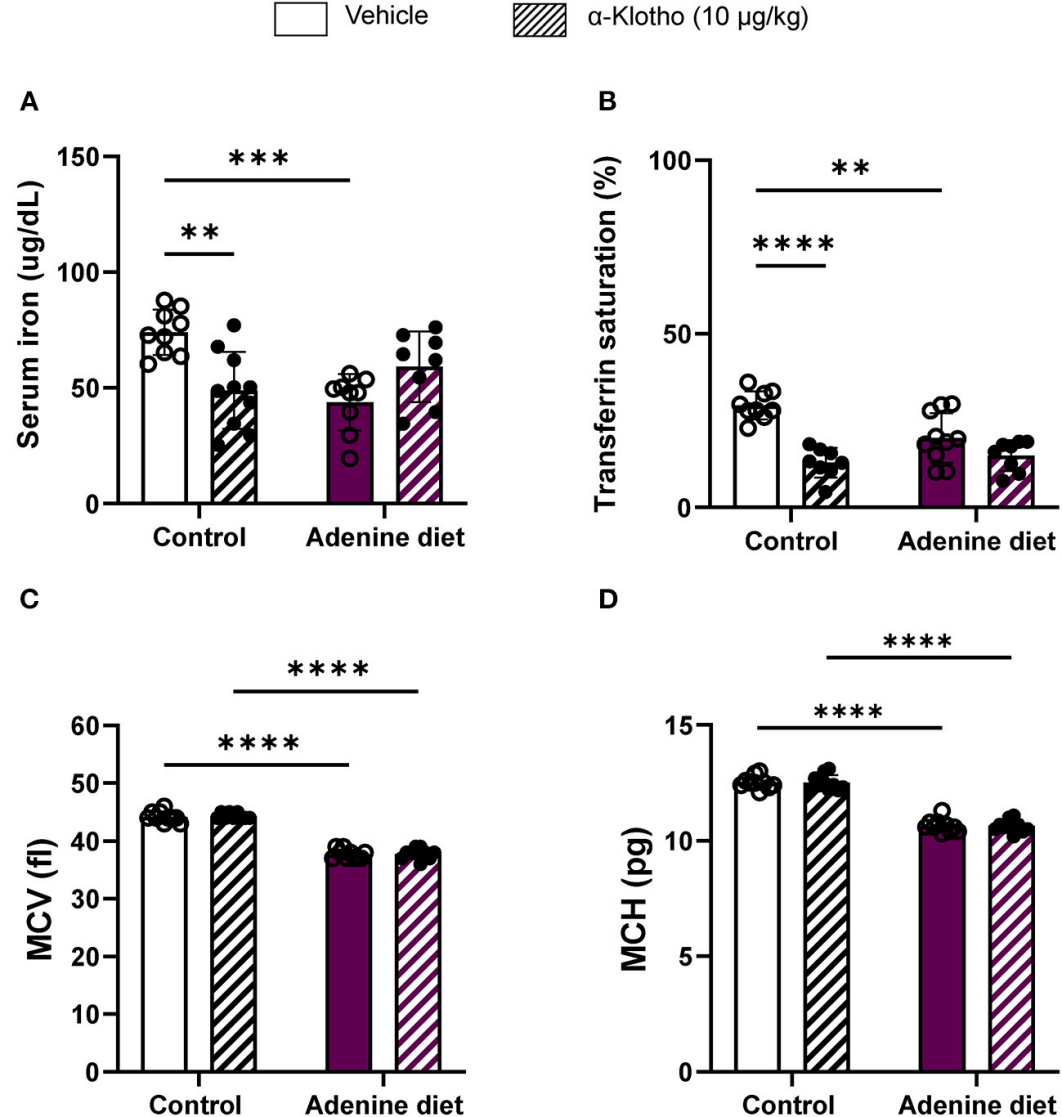
Iron homeostasis in response to CKD and klotho supplementation. C57BL/6J male mice were fed control diet or 0.2% adenine diet. After 8 weeks, mice in each group were further divided into 2 groups and were injected with either saline or mouse recombinant Klotho protein (10 μg/kg) 24 h prior to sacrifice. Serum samples were obtained and iron concentration was determined by colorimetric method. **(A)** Serum iron, **(B)** transferrin saturation. Whole blood was collected separately at sacrifice and quantified for MCV and MCH using Hematology analyzer. **(C)** Mean corpuscular volume (MCV), **(D)** mean corpuscular hemoglobin (MCH). Data are represented as mean ± SD (*n* = 8–10 per group). **P < 0.01, ***P < 0.001, ****P < 0.0001 compared to Control.

Hepcidin inhibits intestinal iron absorption and release of stored iron, thereby limiting available iron to be transported to the circulation and resulting in iron deficiency (51, 52). Iron deficiency is common among CKD patients with anemia characterized by microcytic hypochromic red blood cells (5). Our study confirms the presence of iron deficiency in adenine-fed mice as determined by low serum iron levels (Figure 5A; Table 3) and transferrin saturation (Figure 5B, compared to control mice. Moreover, red blood cells were microcytic and hypochromic, as it was evident by the low mean corpuscular volume (MCV) (Figure 5C; Table 3) and mean corpuscular hemoglobin (MCH) (Figure 5D; Table 3), representing RBC size and amount of hemoglobin per RBC, respectively. Klotho treatment did not affect serum iron or transferrin saturation in adenine-fed mice but it reduced these parameters in control mice (Figures 5A,B). Moreover, Klotho did not affect the size of RBCs or the amount of hemoglobin present in RBCs in either adenine-fed or control mice (Figures 5C,D).

**Figure 6:**
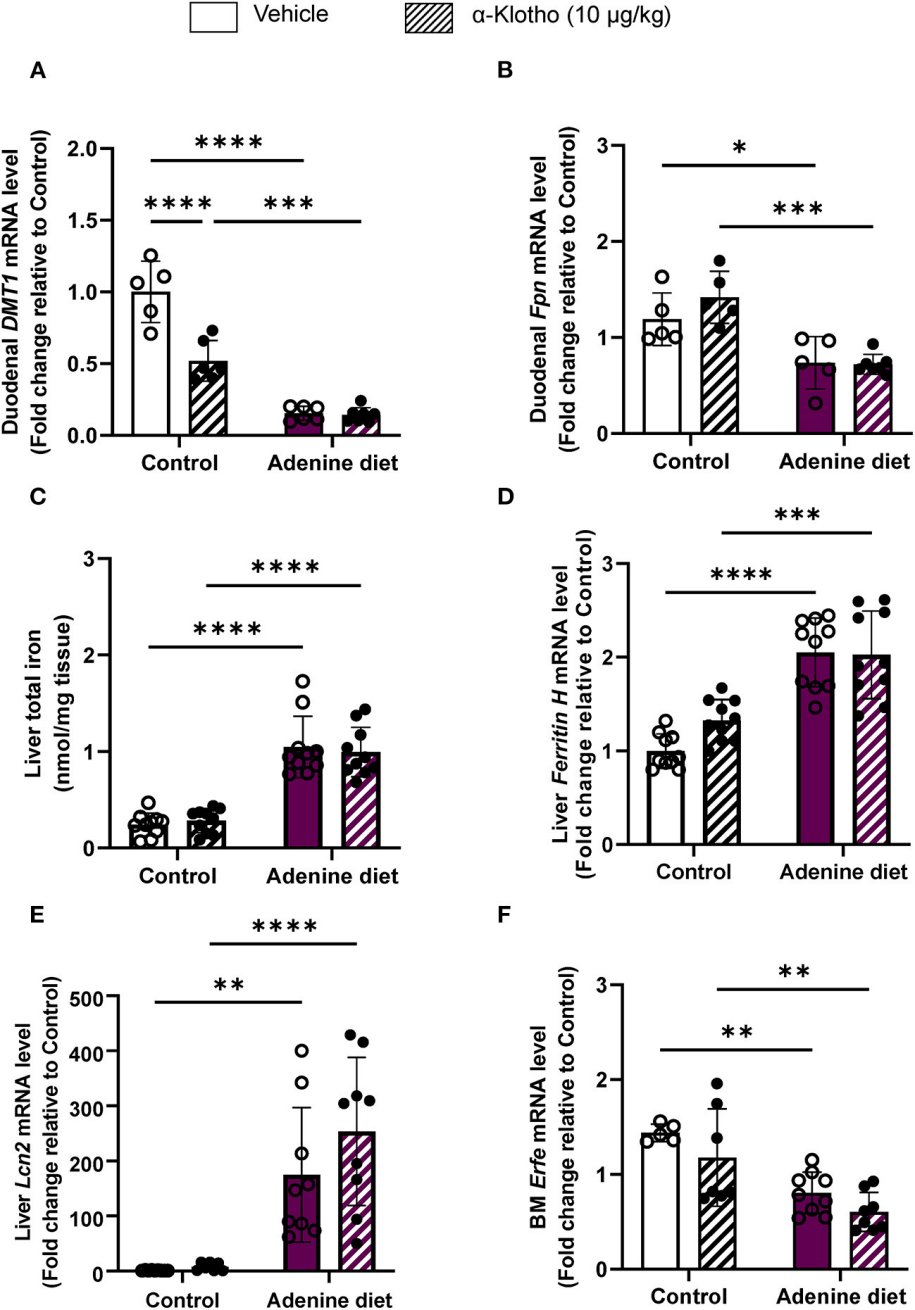
Dysregulation of iron transport and storage in CKD. C57BL/6J male mice fed control or 0.2% adenine diet for 8 weeks were administered with either saline or mouse recombinant Klotho protein (10 ug/kg) 24 h before sacrifice. Quantitative real-time RT-PCR for duodenal **(A)** *Dmt1* and **(B)** *ferroportin* (*Fpn*) mRNA levels (*n* = 5–7 per group). **(C)** Total iron content in the liver by colorimetric assay. Quantitative real-time RT-PCR for **(D)** hepatic *ferritin H* **(E)** hepatic *Lipocalin 2* (*Lcn2*) and **(F)** bone marrow *erythroferrone* (*Erfe*) mRNA expression. qRT-PCR data are expressed as fold change (2^ΔΔCt^) relative to housekeeping gene *Hprt*. Data are represented as mean ± SD (*n* = 8–10 per group). *P < 0.05, **P < 0.01, ***P < 0.001, ****P < 0.0001 compared to Control.

Iron uptake is facilitated by divalent metal transporter 1 (DMT1), whereas iron export is achieved by ferroportin 1 (FPN1). The action of hepcidin is tissue-specific. Studies have shown that hepcidin decreases dietary iron absorption by inhibiting iron transport in enterocytes through downregulation of DMT1 and suppression of ferroportin activity (53–55), whereas in hepatocytes it prevents the mobilization of hepatic iron stores resulting in iron overload (52). In agreement with these data, we found that *DMT1* and *FPN1* are significantly suppressed in the duodenum of adenine-fed mice but did not decrease any further by Klotho (Figures 6A,B). However, Klotho significantly decreased duodenal DMT1 in control mice (Figure 6A). The decrease in serum iron in adenine-fed mice was associated with increased liver iron retention (Figure 6C), which correlated with increased hepatic expression of the iron storage protein ferritin H (*Fth*) (Figure 6D) and the iron sequester lipocalin 2 (*Lcn2*) (Figure 6E). The number of circulating neutrophils, which are the main source of lipocalin secretion, was also increased in adenine-fed mice (Table 3; Supplementary Figure 3). Klotho had no effect in liver iron content (Figure 6C), ferritin (Figure 6D), lipocalin (Figure 6E), or neutrophil number (Supplementary Figure 3) in either CKD or control mice. Moreover, we assessed expression of erythroferrone (Erfe), a hormone produced by bone marrow erythroblasts in response to increased renal EPO production that acts on hepatocytes to suppress hepcidin synthesis, thereby mobilizing iron from stores (56). Our data show that reduced EPO secretion in adenine-fed mice (Figures 3A,B) led to decreased *Erfe* expression in the bone marrow of adenine-fed mice compared to control mice (Figure 6F). Klotho did not affect *Erfe* expression in control or CKD mice (Figure 6F). Taken together, our data demonstrate that mice fed adenine diet show consistent characteristics of renal anemia and iron deficiency, and suggest that iron deficiency in adenine-induced CKD is due to upregulation of hepcidin by inflammation resulting in inhibition of iron absorption and sequestration of iron in liver. Furthermore, Klotho administration did not improve iron deficiency in CKD mice but it decreased intestinal absorption of iron in control mice leading to reduced serum iron and transferrin saturation levels.

## Discussion

Chronic kidney disease (CKD) is an inflammatory condition characterized by impaired excretory capacity and a gradual loss of renal function, putting patients with the condition at a higher risk for cardiovascular disease and mortality. There is no treatment to cure CKD, only to slow it down. Treatment options include medications that help manage symptoms, and in later stages, dialysis or kidney transplant. Complications such as anemia and iron deficiency are associated with poor outcomes such as reduced quality of life and increased cardiovascular disease, hospitalizations, cognitive impairment, and mortality (1). The cause of renal anemia is multifactorial and includes impaired EPO production due to renal dysfunction, pro-inflammatory cytokine activation, and inhibition of the renin-angiotensin system (RAS) by the use of angiotensin converting enzyme (ACE) inhibitors (3). To add to these causes, we previously reported that elevated FGF23 in CKD is significantly contributing to the development of renal anemia (18).

FGF23 signal transduction is achieved by binding of FGF23 to the Klotho-FGFR complex, with Klotho being an obligatory co-receptor for FGF23. *Klotho* is primarily expressed in the kidney and its expression is significantly reduced in CKD (57). Deletion of *Klotho* in mice results in a phenotype that resembles CKD, including hyperphosphatemia, elevated FGF23, ectopic soft tissue calcifications, and decreased plasma and renal Klotho (57, 58). Current treatment for renal anemia is the use of ESAs, which they are associated with a number of adverse outcomes such as worsened hypertension and cardiovascular disease, thromboembolism, tumor growth, and resistance to Epo therapy itself (1, 3, 11–13, 59). In search for treatment options with less adverse effects, we previously reported that inhibition of FGF23 signaling by the use of an FGF23 antagonist successfully rescues renal anemia and attenuates iron deficiency and inflammation in a CKD mouse model (18). Previous studies have reported an association of α-Klotho with several diseases, such as kidney disease (60), cardiovascular disease (61), and periodontitis (62). However, its role in hematological disorders, including anemia, remains unclear. Based on prior studies demonstrating beneficial and therapeutic effects of Klotho administration in CKD (23, 63–65) and our own work showing that disruption of FGF23 signaling by genetic ablation of *Fgf23* or *Klotho* results in increased erythropoiesis (16, 17), in the present study we investigated the effect of exogenous α-Klotho in the development of renal anemia.

Our data show that α-Klotho administration had no beneficial effect in mice with CKD-associated anemia as it did not increase RBC numbers and hemoglobin levels, and it did not stimulate EPO secretion. Moreover, α-Klotho did not improve iron deficiency and inflammation in CKD as it had no effect on iron levels or inflammatory markers. These unexpected data suggest that one bolus injection of 10μg/Kg of recombinant α-Klotho was not sufficient to improve established anemia and iron deficiency in a CKD mouse model. Our choice of the given dose of Klotho (10μg/Kg) was based on published data showing that this dose was sufficient to prevent or delay AKI to CKD progression and uremic cardiomyopathy in mice (63) and resulted in less kidney damage in mice with AKI (32).

However, in agreement with our previous work (17), Klotho significantly decreased the number of bone marrow erythroid progenitors (BFU-E), in mice fed control diet, through downregulation of renal *Hif2*α and subsequent decrease in synthesis of EPO in the kidney and its secretion into the circulation. It also decreased serum iron and transferrin saturation by reducing duodenal iron transport through downregulation of *DMT1* in control mice. Thus, although α-Klotho does not rescue renal anemia and associated iron deficiency, in physiological conditions α-Klotho appears to have an inhibitory effect on erythropoiesis and iron regulation. Our data are consistent with other reports that demonstrated a negative correlation between Klotho and iron and reported that serum iron overload decreases renal mRNA and protein levels of Klotho, whereas iron chelation suppresses angiotensin II-induced downregulation of Klotho (24, 66). Furthermore, in the same study, it was shown that a free radical scavenger suppressed the angiotensin II-induced downregulation of *klotho*, suggesting a role for reactive oxygen species (ROS) production in this process.

**Figure 7:**
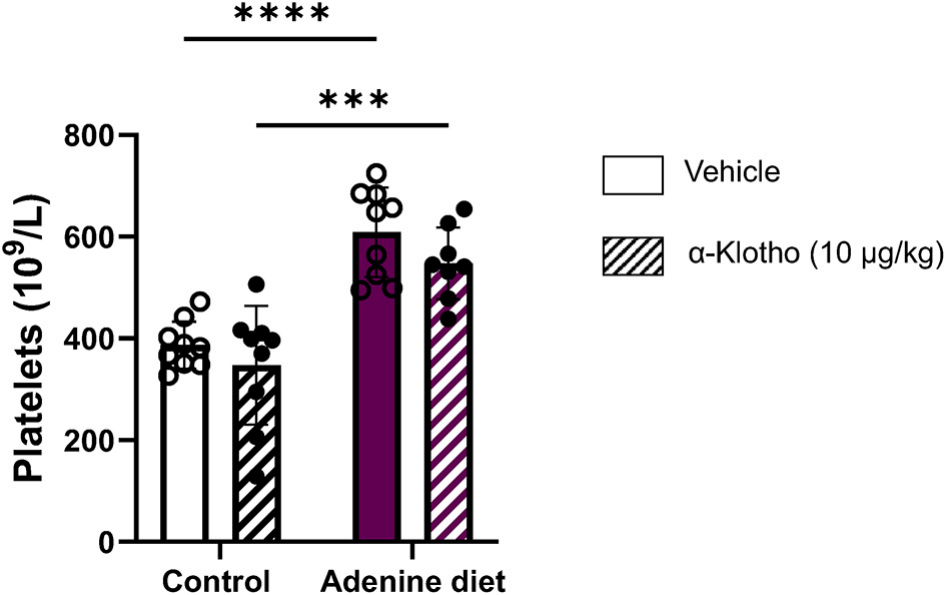
Effect of adenine diet and Klotho administration on platelets. C57BL/6J male mice fed control or 0.2% adenine diet for 8 weeks were administered with either saline or mouse recombinant Klotho protein (10 μg/kg) 24 h before sacrifice. Whole blood was collected and platelet concentration was quantified using Hematology Analyzer. Data are represented as mean ± SD (*n* = 8–9 per group). ***P < 0.001, ****P < 0.0001 compared to Control.

Iron deficiency in CKD is due to decreased iron absorption and increased iron loss caused by GI bleeding or platelet dysfunction (5). Moreover, studies have shown that iron deficiency is associated with elevated number of platelets as it was determined by a negative correlation between platelet count and transferrin saturation in CKD patients (67, 68). Conversely, it has been shown that iron repletion treatment reduces platelet numbers in patients with CKD (67–71). Consistent with these studies, our data show a significantly elevated platelet count in adenine fed mice (Table 3; Figure 7). However, Klotho did not have any effect on platelet numbers in either group (control or adenine) of our mice. The mechanism of platelet elevation in response to iron deficiency is not determined. Previous reports suggest that iron may inhibit platelet maturation (67, 72), whereas iron deficiency may affect platelet production through EPO (67, 73, 74) or have a direct effect on megakaryopoiesis by resulting in megakaryocyte progenitor expansion (67, 75). Since Klotho reduced serum iron and transferrin saturation in our control mice, we expected to see increase in platelet count in these mice. Lack of platelet count elevation in our control mice suggests that iron deficiency stimulates platelet production in the presence of inflammation.

**Figure 8:**
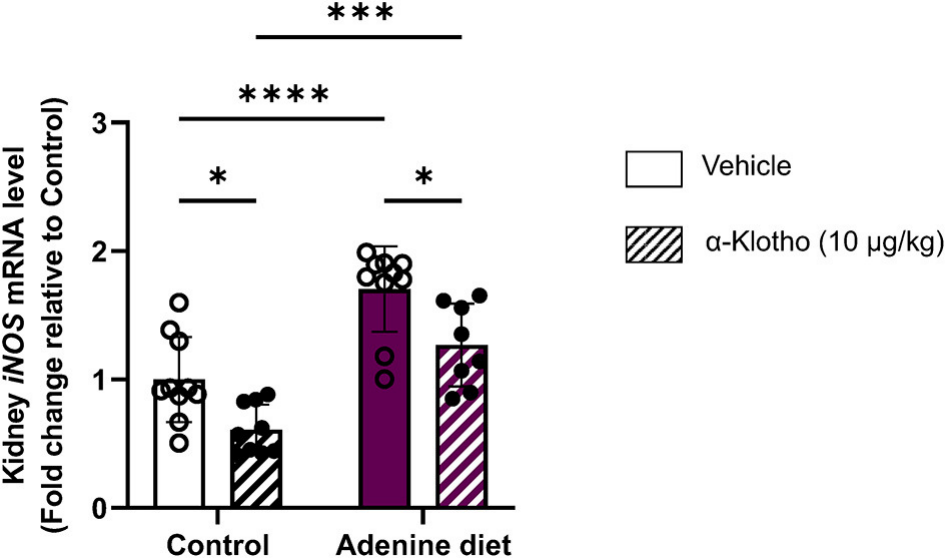
Response of renal iNOS mRNA expression in CKD and klotho administration. C57BL/6J male mice were fed control diet or 0.2% adenine diet. After 8 weeks, mice in each group were further divided into 2 groups and were injected with either saline or mouse recombinant Klotho protein (10 μg/kg) 24 h prior to sacrifice. Quantitative real-time RT-PCR for renal *inducible nitric oxide synthase* (*iNOS*). Data are expressed as fold change (2^ΔΔCt^) relative to housekeeping gene *Hprt*. Samples were measured in duplicates. Data are represented as mean ± SD (*n* = 8–9 per group). *P < 0.05, ***P < 0.001, ****P < 0.0001 compared to Control.

Activation of inducible nitric oxide synthase (iNOS; also referred to as NOS2) is associated with inflammatory conditions such as CKD and results in high levels of nitric oxide (NO) that lead to enhanced oxidative damage by increased generation of ROS (76–79). Thus, high levels of NO produced by iNOS may contribute to the progression of renal disease (79). It has been shown that the use of iNOS inhibitors may block the activation of pro-inflammatory cytokines produced during inflammation, resulting in translocation of NF-κB from cytoplasm to the nucleus and increase of *iNOS* expression (80). In agreement with these reports, our data show increased iNOS expression in adenine fed mice compared to control mice (Figure 8). Klotho exerts anti-inflammatory effects and negatively regulates the production of NF-κB-associated inflammatory proteins (81). Moreover, in CKD Klotho may mitigate activation of NF-κB by downregulating the expression of toll-like receptor 4 (TLR4) (82, 83). Conversely, it has been reported that NF-κB suppresses the activity of Klotho by possibly promoting ROS production (84). In agreement with its anti-inflammatory role, Klotho treatment significantly reduced *iNOS* expression in the kidney in both control- and adenine-fed mice (Figure 8).

Our study has a number of limitations. First, the lack of a standardized assay to measure plasma Klotho, rendered us unable to confirm reduced Klotho levels in the adenine mice and assess the degree of circulating Klotho increase after α-Klotho administration. Second, our choice to use the adenine CKD mouse model vs. the 5/6 nephrectomy (Nx) mouse model may have played a role in the outcome of our study. Although the duration of disease progression is similar between the two mouse models (adenine and 5/6 Nx), the adenine diet model produces rapid-onset kidney disease with extensive tubulointerstitial fibrosis and tubular atrophy, whereas in the 5/6 Nx model the remaining kidney is functional and assumes a heavy-duty filtration function resulting in eventual nephron damage and occurrence of CKD. In addition, we previously showed that inhibition of FGF23 signaling rescues renal anemia and iron deficiency in mice with CKD induced by 5/6 nephrectomy (18). One important difference between our published study (18) and the present adenine study is the degree of increase in FGF23 levels. In the 5/6 Nx model, FGF23 levels were increased ~2.5 times above normal levels (306.3 vs. 801.2 pg/ml) (18), whereas in the present adenine study, FGF23 levels were massively increased ~13 times above normal levels (293.287 vs. 3911.919 pg/ml). Thus, the difference in circulating FGF23 and in the form of decreased renal function may have had important impact on whether Klotho injections alter FGF23-mediated changes on red blood cell production and iron regulation. Third, it is possible that we did not see any effect of Klotho in adenine-fed mice due to the chosen dose and duration of Klotho injection. In the present study, we injected mice with 10 μg/Kg of recombinant α-Klotho, a dose similar to what other investigators used in published reports (32, 63). In these published studies, investigators showed that this dose is sufficient to prevent or delay AKI to CKD progression and uremic cardiomyopathy in mice (63) and results in less kidney damage in mice with AKI (32). Our decision to use 10μg/Kg of recombinant α-Klotho was also based on our previous published work where we injected the same dose and we observed significant effects on the hematopoietic phenotype of wild-type mice (17). However, it is possible that one bolus injection at this dose was not sufficient to see an effect in adenine-fed mice in which Klotho levels are already low. Consideration should be given to assessing the effect of klotho after 3–4 daily injections (acute effect) or using minipumps delivering Klotho over a few weeks for a chronic effect, as described by Hu et al. (63). A dose and time course would be necessary to assess the effects of exogenous Klotho on hematological and iron parameters. One other aspect to consider, is the half-life of circulating Klotho. Hu et al. showed that although the levels of circulating exogenous Klotho immediately after injection were similar between normal mice and mice with CKD, the half-life of circulating exogenous Klotho (10 μg/Kg) in mice with CKD was much longer than in normal mice (25 h vs. 7.2 h) and similar to the levels of endogenous Klotho in mice with CKD (26.6 h) (14). These results show that normal healthy kidneys clear circulating Klotho faster and also suggest that the reason we did not see any increase on renal Klotho expression after administration of exogenous Klotho is because excess Klotho was already cleared by the kidneys after 24 h.

Because of the adverse effects ESAs have, it is clear that there is a need for treatment options that have fewer side effects. Recently, Hanudel et al. showed that Vadadustat, a hypoxia-inducible factor-prolyl hydroxylase inhibitor (HIF-PHI), ameliorates CKD-associated anemia and improves iron levels, as well as reducing the loss of kidney function and lowering FGF23 (85). Our previous work also clearly demonstrated the efficiency of inhibiting FGF23 signaling in stimulating erythropoiesis and rescuing anemia, iron deficiency and inflammation (18). Moreover, we found that blocking FGF23 signaling significantly increased renal Klotho expression in mice with CKD induced by 5/6 nephrectomy. Therefore, FGF23 antagonists are strong candidates for the treatment of renal anemia, iron deficiency and associated inflammation, and have the potential to attenuate Klotho deficiency. Although in our study one bolus injection of recombinant Klotho at the chosen dose did not improve anemia and iron deficiency in CKD, it does not exclude the possibility that suppression of Klotho levels deteriorates CKD-associated anemia and, thus, optimization of the treatment protocol may provide evidence that Klotho may still be a potential therapeutic target for renal anemia.

## Data Availability Statement

The original contributions presented in the study are included in the article/Supplementary Material, further inquiries can be directed to the corresponding author.

## Ethics Statement

The animal study was reviewed and approved by New York University IACUC.

## Author Contributions

MP performed research, analyzed data, assisted with data interpretation, and manuscript preparation. CLH assisted with experimental procedures. DS designed and performed research, analyzed data, wrote the manuscript, and oversaw the study. All authors contributed to the article and approved the submitted version.

## Funding

This work was supported by funds from the US Department of Defense (W81XWH-16-1-0598) to DS.

## Conflict of Interest

The authors declare that the research was conducted in the absence of any commercial or financial relationships that could be construed as a potential conflict of interest.

## Publisher's Note

All claims expressed in this article are solely those of the authors and do not necessarily represent those of their affiliated organizations, or those of the publisher, the editors and the reviewers. Any product that may be evaluated in this article, or claim that may be made by its manufacturer, is not guaranteed or endorsed by the publisher.
