# Supplementary material for: Administration of α-Klotho Does Not Rescue Renal Anemia in Mice
Source: Front Pediatr. 2022 Jun 23;10:924915. doi: 10.3389/fped.2022.924915 (PMC9259788; doi:10.3389/fped.2022.924915)
Supplement: Supplementary file 1 [file Data_Sheet_1.PDF]

# Supplementary Table 1

**Supplementary Table 1. List of the primers used in the study**

| Gene | Primer | Sequence (5' - 3') |
|---|---|---|
| *Klotho* | Forward primer | AAA TGG CTG GTT TGT CTC GGG AAC |
| | Reverse primer | TAT GCC ACT CGA AAC CGT CCA TGA |
| *NaPi2a* | Forward primer | GTG CCT CTG ATG CTG GCT TTC |
| | Reverse primer | CTG GAA CTC TGC ACC AGA ACT |
| *NaPi2c* | Forward primer | CTC ACC ATA CAT GCA GAG CTA GGA |
| | Reverse primer | TGC ATT TCT CAG ACT CCG GT |
| *Epo* | Forward primer | TCT ACG TAG CCT CAC TTC ACT |
| | Reverse primer | ACC CGG AAG AGC TTG CAG AAA |
| *Erfe* | Forward primer | GGG CTG CAT GGA CAA ACT |
| | Reverse primer | GCC GCT TTG CTC TCA AAC TT |
| *Hif2α* | Forward primer | GGG AAC ACT ACA CCC AGT GC |
| | Reverse primer | TCT TCA AGG GAT TCT CCA AGG |
| *Tnfα* | Forward primer | AAG GGA GAG TGG TCA GGT TGC C |
| | Reverse primer | CCT CAG GGA AGA GTC TGG AAA GG |
| *IL-6* | Forward primer | ATC CAG TTG CCT TCT TGG GAC TGA |
| | Reverse primer | TAA GCC TCC GAC TTG TGA AGT GGT |
| *Hepcidin (Hamp)* | Forward primer | CAC CAC CTA TCT CCA TCA ACA G |
| | Reverse primer | GTT GGT GTC TCT CTT CCT TCT C |
| *Dmt1* | Forward primer | TCA TGG AGG GAT TCC TGA AC |
| | Reverse primer | TCC TCC AGC CTA TTC CAT TG |
| *Fpn* | Forward primer | CTC TGT CAG CCT GCT GTT TG |
| | Reverse primer | TCA GGA TTT GGG GCC AAG ATG |
| *Lipocalin2 (Lcn2)* | Forward primer | CCA GTT CGC CAT GGT ATT TT |
| | Reverse primer | TCC TTC AGT TCA GGG GAC AG |
| *Ferritin (FtH)* | Forward primer | AAG TGC GCC AGA ACT ACC AC |
| | Reverse primer | CAG AGC CAC ATC ATC TCG GT |
| *iNOS* | Forward primer | CCC TTC CGA AGT TTC TGG CAG CAG C |
| | Reverse primer | GGC TGT CAG AGC CTC GTG GCT TTG G |
| *Hprt* | Forward primer | AAG CCT AAG ATG AGC GCA AG |
| | Reverse primer | TTA CTA GGC AGA TGG CCA CA |

# Supplementary Figure 1

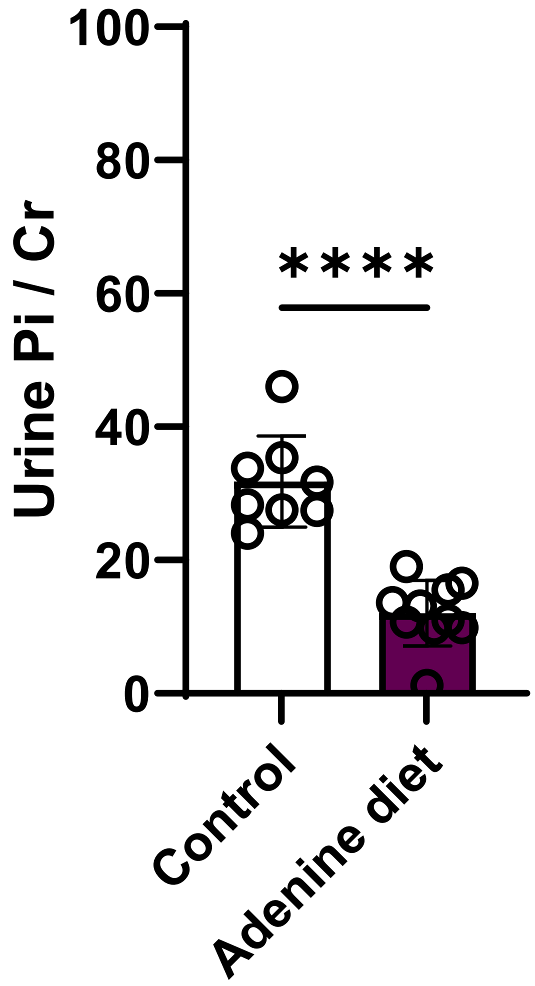


**Supplementary Figure 1. Urinary phosphorus to creatinine ratio**

C57BL/6J male mice fed control or 0.2% adenine diet for 8 weeks. Urine was collected upon anesthesia at sacrifice, and concentration of urinary phosphorus and creatinine were determined by colorimetric assay. Data are represented as mean ± SD. (n=8-10 per group). ****$P < 0.0001$ compared to Control.

# Supplementary Figure 2

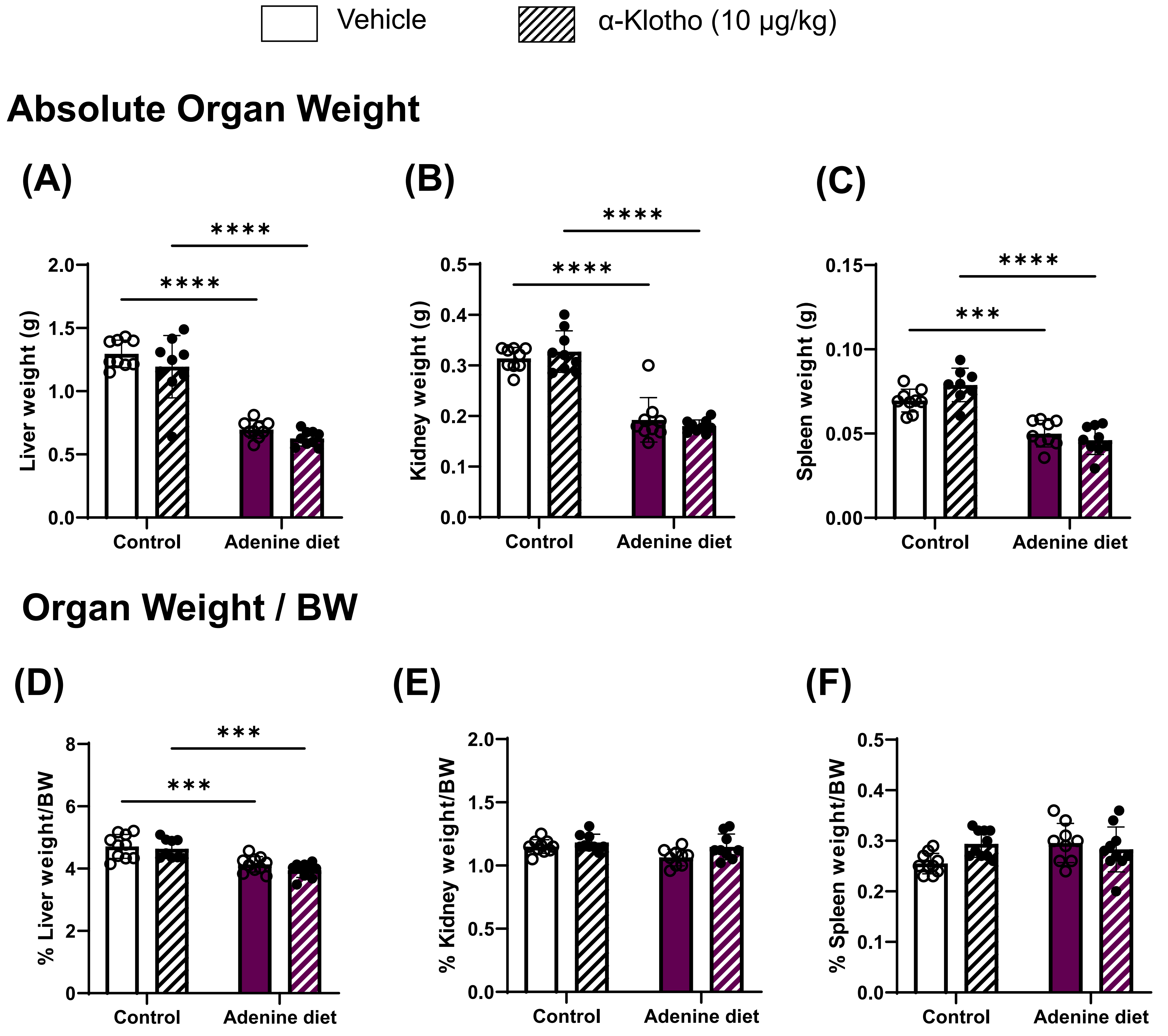


**Supplementary Figure 2. Absolute liver, kidney and spleen weight and organ weight normalized to body weight**

C57BL/6J male mice fed control or 0.2% adenine diet for 8 weeks were administered with either saline or mouse recombinant Klotho protein (10 μg/kg) 24 hours before sacrifice. Absolute organ weight **(A-C)**. **(A)** Liver, **(B)** Kidney, **(C)** Spleen. Percentage of organ weight per body weight (BW) **(D-F)**. **(D)** Liver weight/BW, **(E)** Kidney weight/BW, **(F)** Spleen weight/BW. Data are represented as mean ± SD. (n=7-10 per group). ***$P$ < 0.001, ****$P$ < 0.0001 compared to Control.

# Supplementary Figure 3

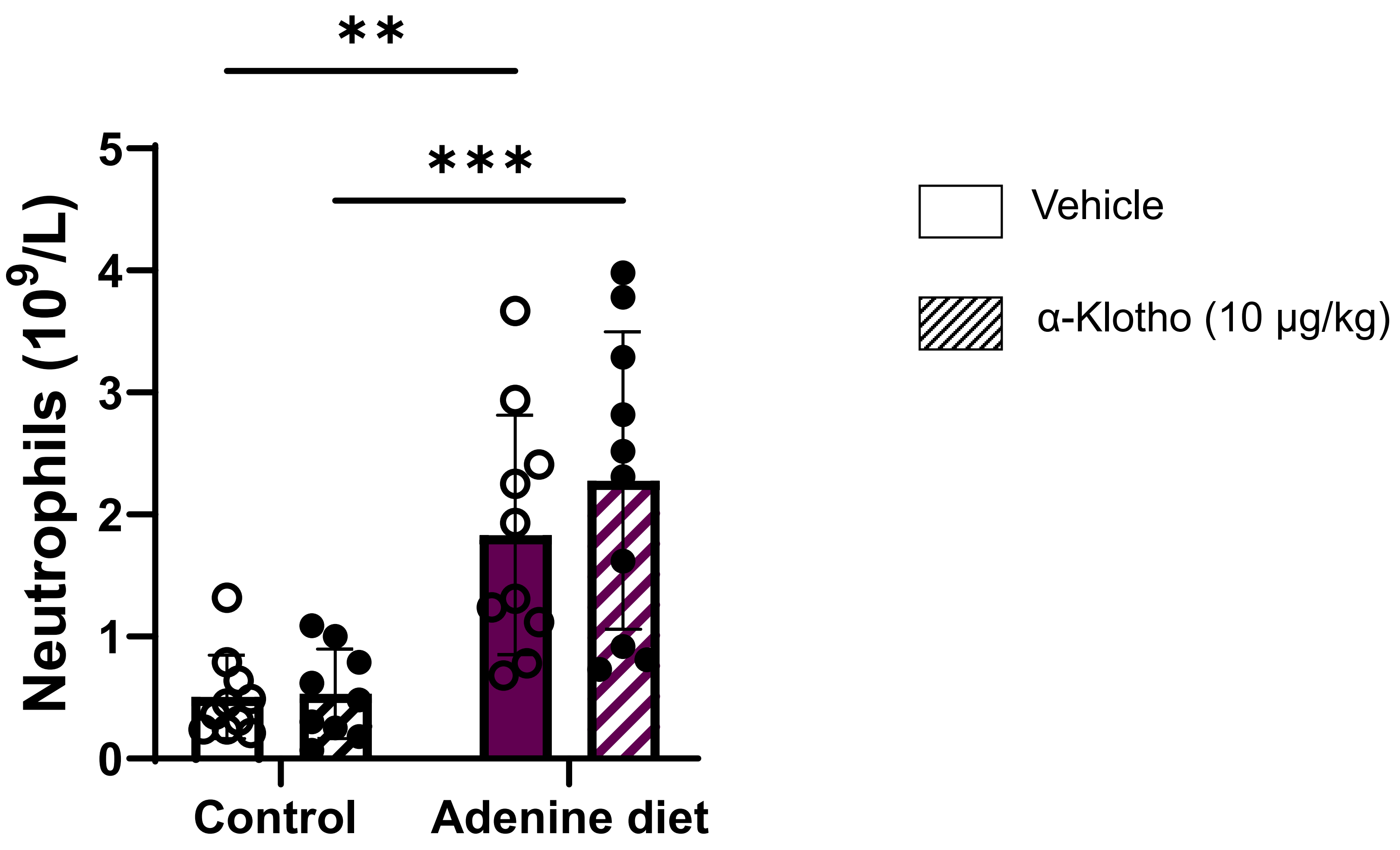


**Supplementary Figure 3. Effect of adenine diet and Klotho administration on neutrophil**

C57BL/6J male mice fed control or 0.2% adenine diet for 8 weeks were administered with either saline or mouse recombinant Klotho protein (10 μg/kg) 24 hours before sacrifice. Whole blood was collected and neutrophil count was quantified using Hematology Analyzer. Data are represented as mean ± SD. (n=8-9 per group). $^{**}P < 0.01$, $^{***}P < 0.001$ compared to Control.
